# Biofilm formation ability and swarming motility are associated with some virulence genes in *Proteus mirabilis*

**DOI:** 10.1186/s12866-025-04090-5

**Published:** 2025-07-02

**Authors:** Mahin Veisi, Hossein Hosseini-Nave, Omid Tadjrobehkar

**Affiliations:** Department of Medical Bacteriology & Virology, Afzalipour School of Medicine, Kerman University of Medical Sciences, 22 Bahman Blvd, Pajoohesh Sq, Kerman, Iran

**Keywords:** *Proteus mirabilis*, Virulence associated genes, Biofilm formation, Swarming motility, *MrpA* gene, *HlyA* gene

## Abstract

*Proteus mirabilis (P. mirabilis)* is one of the frequent causes of urinary tract infection in humans. This pathogen armed by diverse virulence associated factors. Biofilm formation and swarming motility are two surface living behaviors of *P. mirabilis* and their association with virulence associated genes was investigated in the present study. Biofilm formation ability and swarming motility were evaluated by microtiter plate assay and top-agar travel tracking in 91 *P. mirabilis* isolates respectively. The polymerase chain reaction method was used for screening of 10 virulence associated genes. Association of virulence associated genes with biofilm formation ability and also swarming motility was analyzed statistically. The *zapA *(100%) and *hlyA *(41.8%) genes had maximum and minimum frequency respectively. Forty-one, 35 and 15 isolates were categorized as weak, intermediate and strong biofilm producers respectively. While, 11%, 38.5% and 50.5% of isolates were grouped as weak, intermediate and strong swarmers respectively. Adhesin encoding genes such as *mrpA* were more prevalent in strong biofilm producers in comparison to the other isolates. Reversal association of *rsmA gene* with swarming motility was detected. The frequency of *hlyA gene* was associated directly with swarming motility and in opposite way with biofilm formation. Reverse correlation of biofilm formation ability and swarming motility was estimated. Based on the study findings it is hypothesized that *P. mirabilis* benefited from adhesins such as MR/P fimbriae for production of biofilm and successful colonization and then they shift from biofilm formers to strong swarmers in order to reach deeper urinary organs and HlyA toxin is used to overcome the immune system cells. However, it has to confirmed trough future studies.

## Introduction

*Proteus* is a Gram-negative rod that was reported first time by Hauser in 1885 [1]. Proteus genus is classified in Enterobacteriaceae and contains several species. *Proteus mirabilis (P. mirabilis)* is well known bacterial species that is involved in different kind of Human infections including urinary tract infections (UTIs), respiratory tract infections, soft tissue infections, skin and wound infections and even sepsis [2–4]. *Proteus* species armed by many virulence factors including diverse adhesions, hemolysins, endotoxin, swarming motility, urease activity, biofilm formation, iron acquisition systems and immune evasion associated proteins [3]. Multiple fimbriae were introduced as the most important adhesions from *Proteus* [5]. The fimbriae are known as effective factors in biofilm formation and also colonization of *P. mirabilis* and its colonization into the host tissue [6]. The five most investigated fimbriae are mannose *Proteus* like(MR/P) fimbriae, *Proteus mirabilis* fimbriae (PMF), ambient temperature fimbriae (ATF), *Proteus mirabilis* fimbriae (PMF) and uroepithelial cell adhesin (UCA) that also is known as non-agglutinating fimbriae (NAF) [7]. Association of some fimbrial genes such as *mrpA*, *ucaA* and *pmfA* with biofilm formation ability was reported formerly [8, 9]. Two hemolysins HpmA and HlyA have reported as the major toxins from Proteus [10]. The *rsmA* gene in *Proteus mirabilis* had some homolog genes in other bacteria such as *csrA* in *Escherichia coli* and majority of these genes have regulatory activity on some metabolic pathways, motility and even biofilm formation [11]. The *fliL* gene is encoding a flagellar protein in basal body of flagellum in Proteobacteria and involved in regulation of stator activity in this region [12].

Ability of *Proteus* species to grow in single or multispecies communities in biofilm structures has confirmed its role in virulence, survival and pathogenesis of this bacterium especially in UTIs. *P. mirabilis* is reported from less than 2% of community acquired UTIs and also 5% of hospital acquired UTIs [13]. *P. mirabilis* also is one of the leading cause of catheter associated urinary tract infections (CAUTIs) that could complicated with urinary stone formation and blockage of the catheter [14]. *P. mirabilis* is naturally resistant against nitrofurans, tetracyclines, tigecycline and polymyxins and decreased susceptibility against cephalosporins and carbapenem agents also were reported frequently [15]. Many cell surface compartments such as lipopolysaccharide, diverse fimbriae, surface hydrophobicity and even flagellar motility plays a role in different stages of biofilm formation process [6, 8, 16]. Microbial biofilms estimated to play roles in 80% of all human infections. High resistance of pathogens in biofilm matrix against antimicrobial agents and also immune system activities were reported recently [17].

*P. mirabilis* is well known for its prominent swarming motility and biofilm formation that those were reported as obvious surface living behaviors of many pathogenic bacteria. Swarmer cells are elongated 5 to 40 folds and armed by huge number of flagella [18]. Amazing properties of *P. mirabilis* for shape-shifting from short, hypo-flagellated swimmer cells to the long, hyper-flagellated swarmer cells enable them to migrate across the surface efficiently and access environments rich of needed nutrients and also it is associated with the virulence of bacterium [9, 18, 19]. Migration of *P. mirabilis* swarmer cells on the agar media and their periodically differentiation to the normal swimmer cells is resulted to appearance of some concentric circles on the agar surface [18]. Critical role of biofilm formation ability and swarming motility in different steps of *P. mirabilis* pathogenesis including maintenance of bacteria in urinary system, protection against immune system and antimicrobial agents and successful migration of *P. mirabilis* on catheter surface toward the uroepithelium were reported before [20].

In spite of few studies that were performed formerly in order to evaluate the associations between biofilm formation ability and also swarming motility of *P. mirabilis* with different virulence associated genes, many aspects of these associations are unknown. Therefore, the present study designed to reveal such associations and if it reaches trustable results then it could introduce suitable targets for future anti-virulent therapy at next step. Such drug targets may propose revision of current treatment strategies specially against complicated biofilm associated UTIs or transmission of *P. mirabilis* using swarming motility in different devise associated infections.

## Results

### Frequency of virulence associated genes

**Fig. 1:**
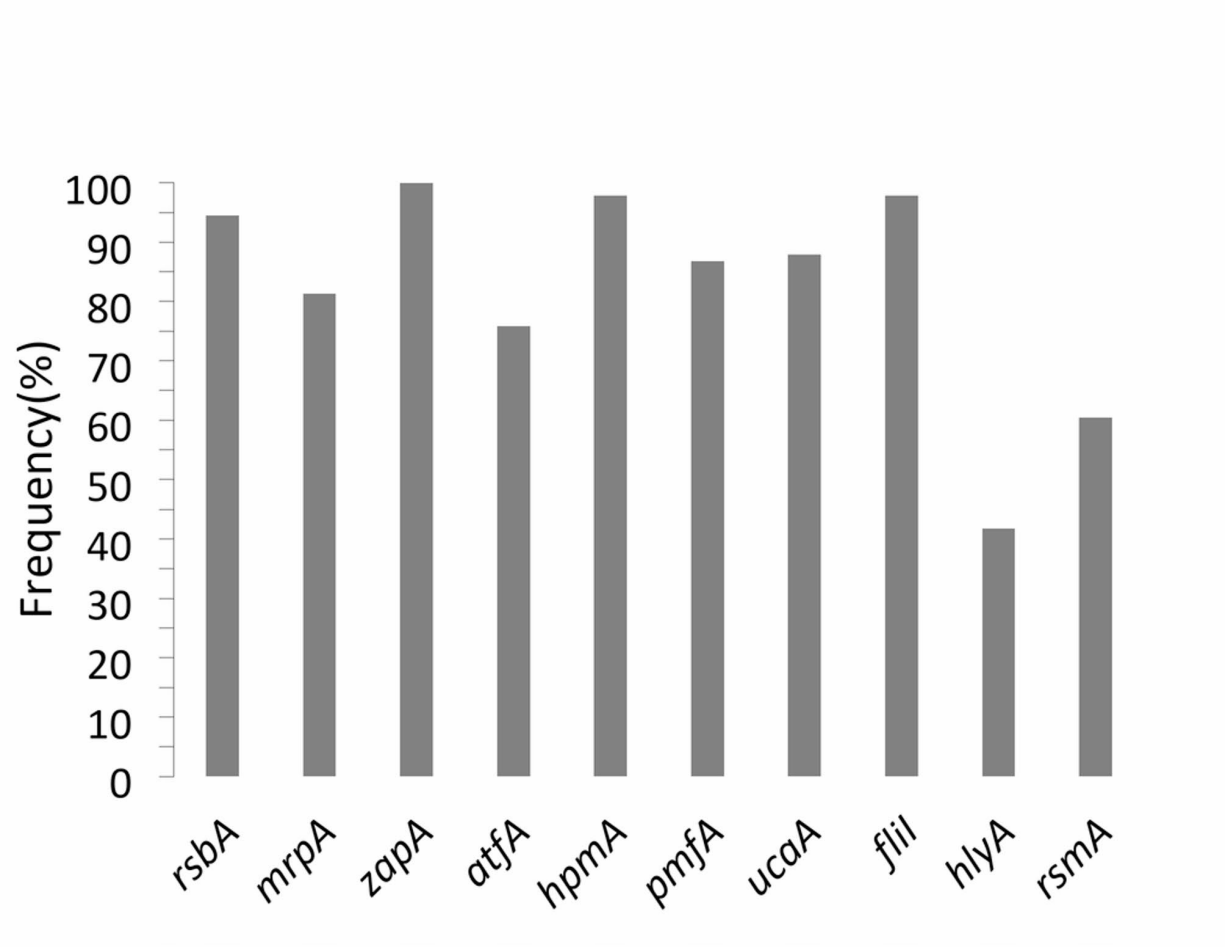
Frequency of different VAGs among *P. mirabilis* isolates

All studied VAGs were found in studied isolates with diverse frequency. The *zapA *(100%) was most prevalent gene and *hlyA *(41.8%) gene had minimum frequency (Fig. 1). Twelve (13.2%) isolates out of 91 isolates had all studied VAGs. Adhesin genes(*ucaA*,* mrpA*,* pmfA* and *atfA*) were detected in 45 (49.5%) isolates.

### Biofilm formation ability

**Fig. 2:**
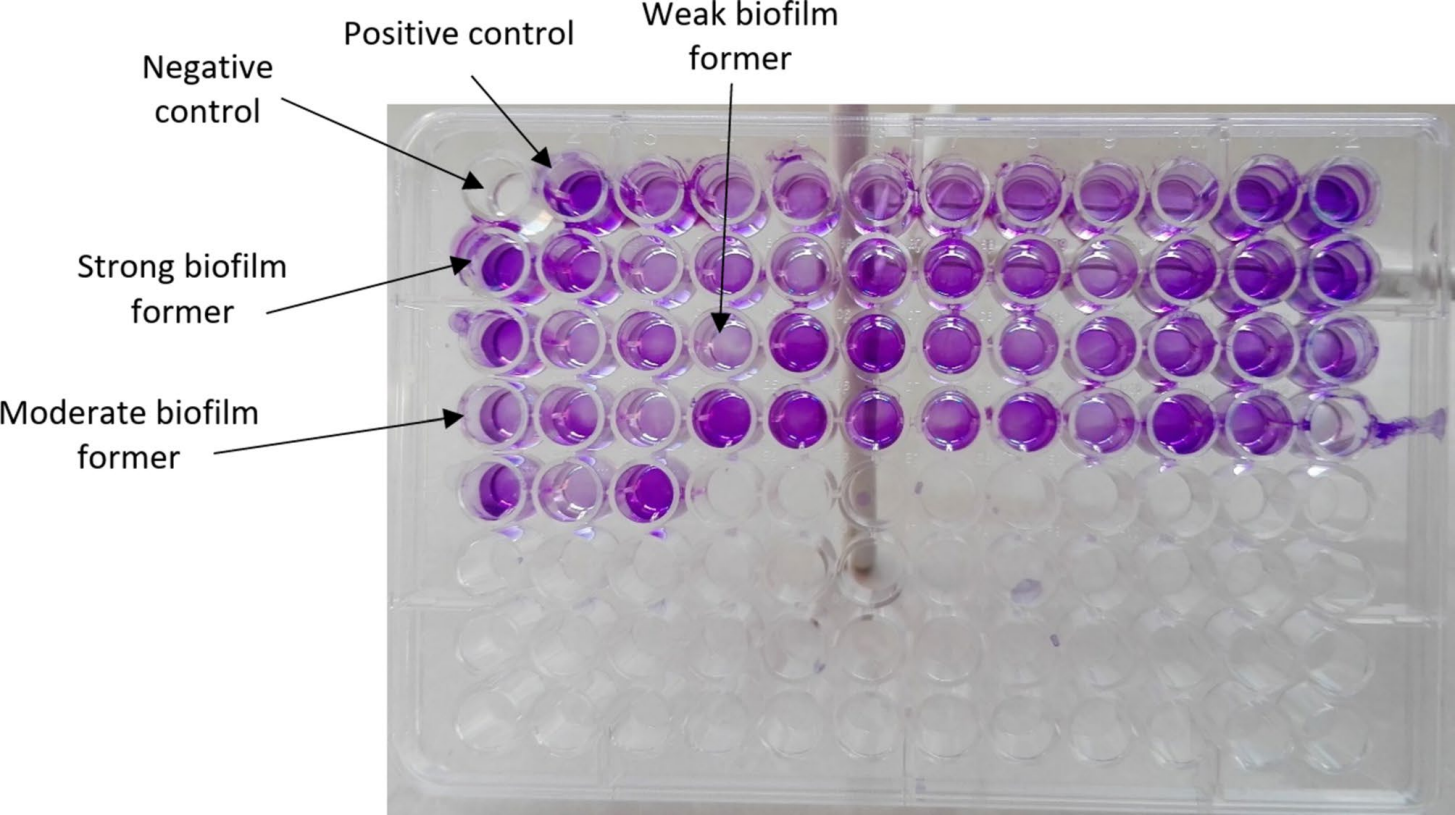
Biofilm formation assessment by microtiter plate test. Strong biofilm formers, moderate biofilm formers and weak biofilm formers were observed in comparison to no-biofilm condition (negative control = broth medium enriched with 1% glucose)

Data analysis showed that all isolates were biofilm producers. In a way that, 41 (45.5%), 35 (38.5%) and 15 (16.5%) were categorized as weak, intermediate and strong biofilm producer respectively (Fig. 2).

### Swarming motility

**Fig. 3:**
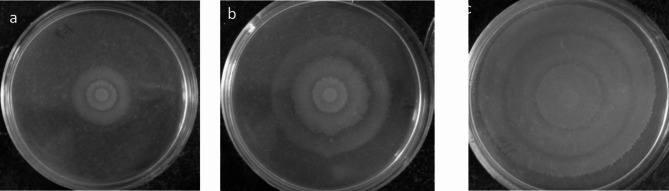
Swarming motility on the agar plate after 18 h incubation period at 37 °C. **a** weak swarmer, **b** intermediate swarmer and **c** strong swarmer

Swarming motility was detected in all studied isolates. Swarming measurements (on the agar surface) revealed a range of 1–8 cm (centimeter) swarming motility among the studied isolates. The most frequent swarming value was 5 cm that was observed in 12 isolates (13.2%). Mean swarming motility was 5.2 cm. Swarming values below 2.7 cm were regarded as weak, values ranging from 2.8 cm to 5.3 were regarded as intermediate swarming and the higher values were regarded as strong swarming motility. Observations revealed, 10 isolates (11%) were regarded as weak swarmer and 35 isolates (38.5%) were regarded as intermediate swarmer and 46 isolates (50.5%) were regarded as strong swarmers (Fig. 3).

**Fig. 4:**
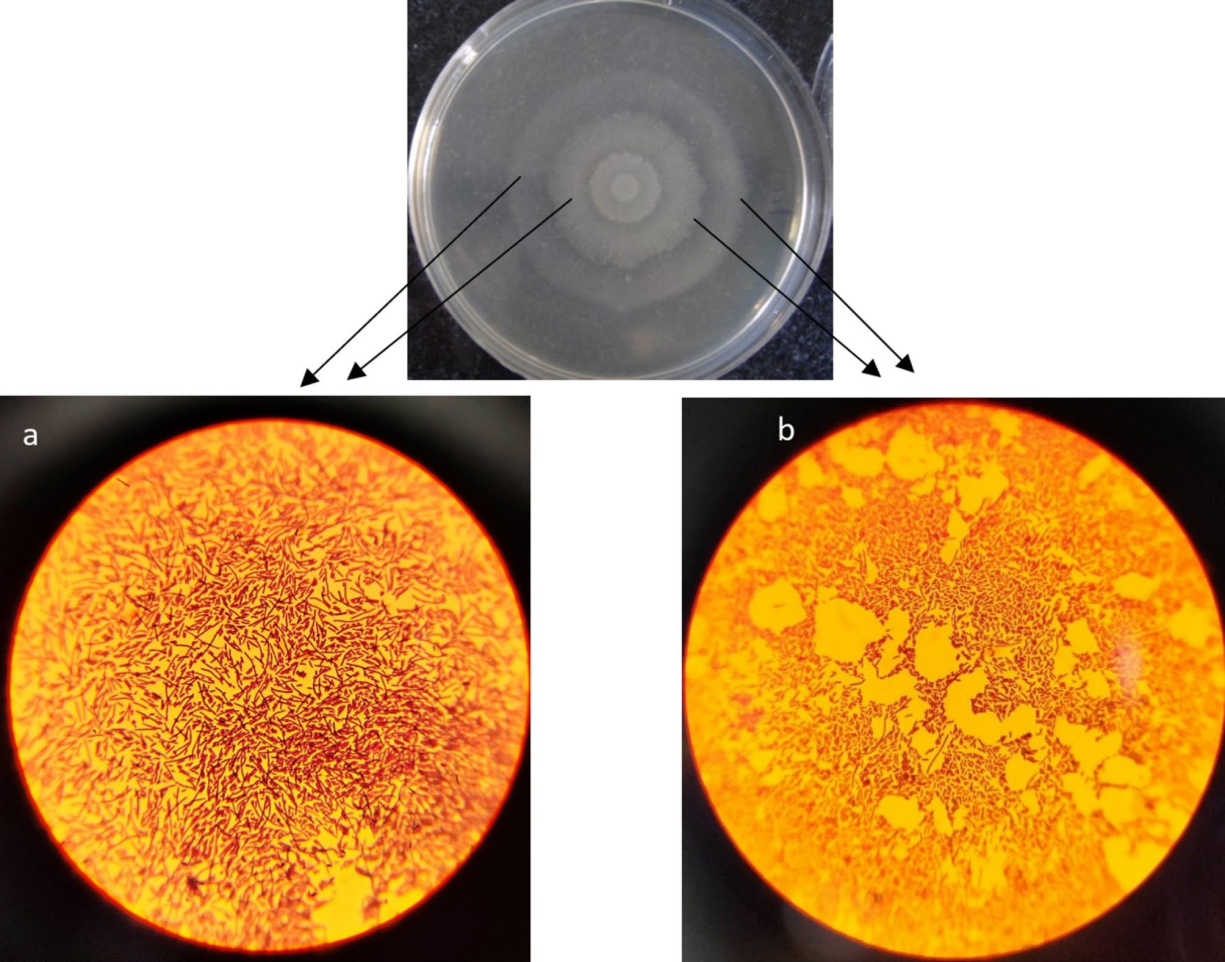
Gram-stained smears from concentric circles in swarming phenomenon. **a** swarmers zone, **b** swimmers zone

Smears stained from different zone of concentric circles of swarming phenomenon showed that bacteria from swarmer zone were significantly longer than bacteria in swimmer zone (Fig. 4).

### Prevalence of studied VAGs among *P. mirabilis* isolates with various biofilm formation ability

**Fig. 5:**
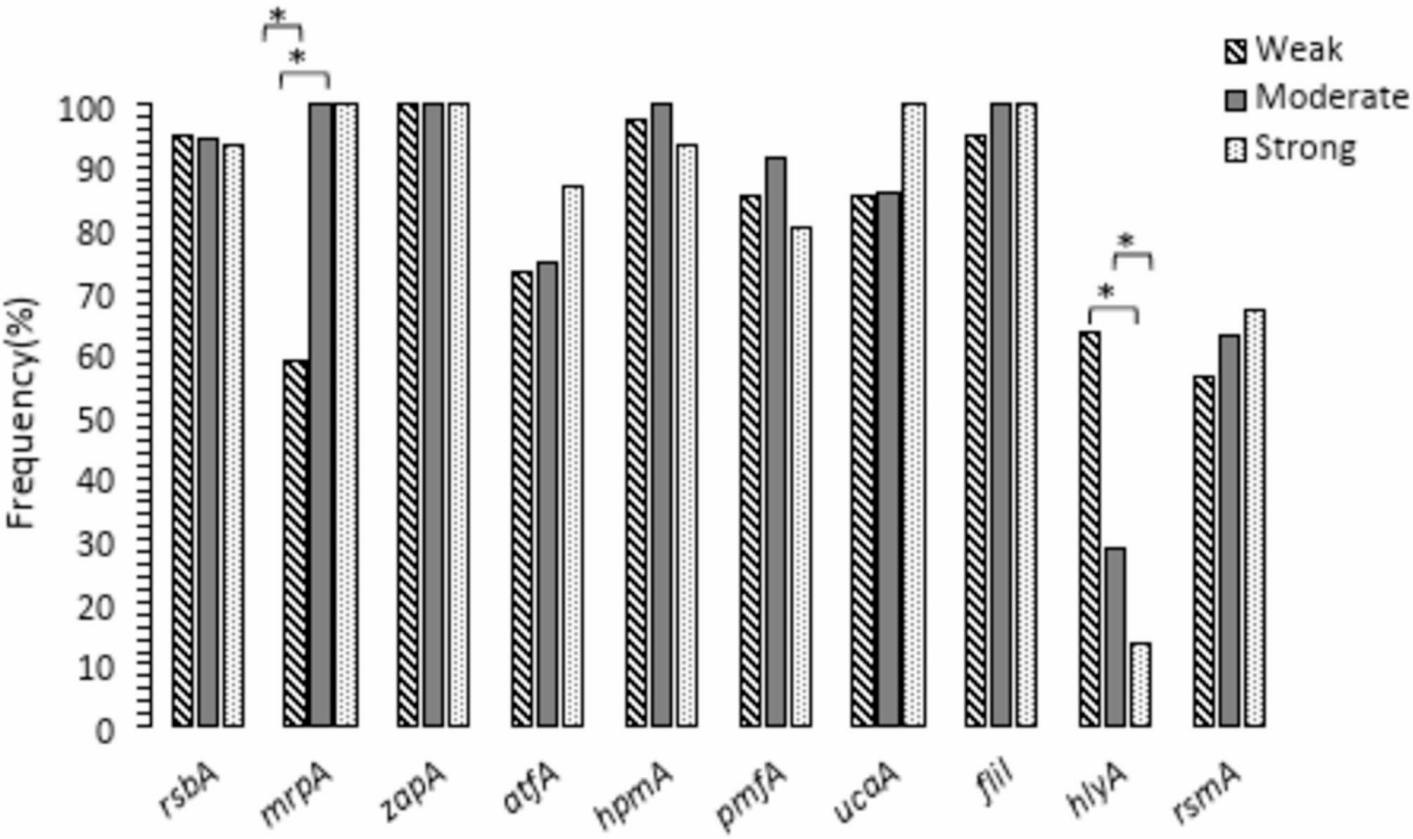
Frequency of different VAGs regarding biofilm formation ability among *P. mirabilis* isolates. *p < 0.001

Chi-square analysis showed frequency of *hlyA* and *mrpA* genes were significantly different (*p* ≤ 0.001) among isolates with different biofilm formation ability. The *hlyA* gene was detected among 2 (13.3%) of strong biofilm formers, while it was found in 26 (63.4%) and 10 (28.6%) of weak biofilm formers and intermediate biofilm formers respectively. Frequency of *mrpA* gene was 24 (58.5%), 35 (100%) and 15 (100%) among weak, moderate and strong biofilm formers respectively. Frequency of other VAGs were not significantly different regarding biofilm formation ability (Fig. 5).

Logistic regression analysis showed *hlyA* gene is a predictor for weak biofilm formation ability (*p* = 0.001 and OR = 6.477).

### Correlation of biofilm formation ability with swarming motility in *P. mirabilis* isolates

**Fig. 6:**
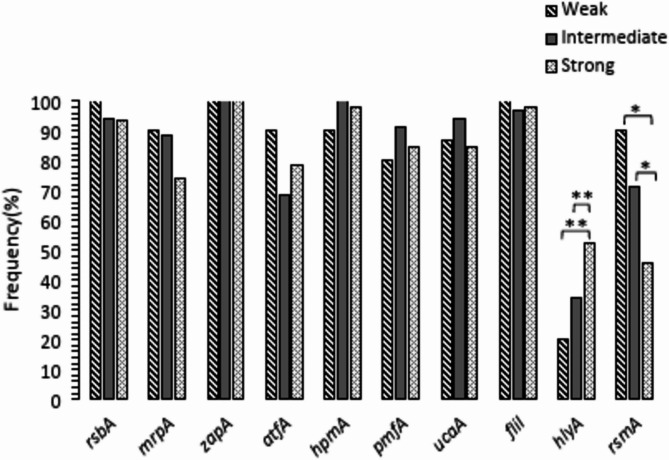
Frequency of different VAGs regarding swarming motility among *P. mirabilis* isolates. *p < 0.01. **p < 0.05

Chi-square analysis showed *rsmA* gene was significantly (*p* = 0.008) less prevalent (45.7%) among strong swarmers in comparison to the intermediate (71.4%) and weak (90%) swarmers. But *hlyA* gene was significantly (*p* = 0.043) more prevalent in strong swarmers (52.2%) in comparison to intermediate swarmers (34.3%) and weak swarmers (20%) Frequency of other studied VAGs were not significantly different among the isolates with different swarming motility (Fig. 6).

Predictory role of *rsmA* gene for weak swarming motility was detected by Logistic regression analysis (*p* = 0.042 and OR = 21.169). Logistic regression analysis was also showed the predictory role of *hlyA* gene for strong swarming motility (*p* = 0.046 and OR = 2.335).

### Correlation of biofilm formation ability with swarming motility in studied isolates

**Fig. 7:**
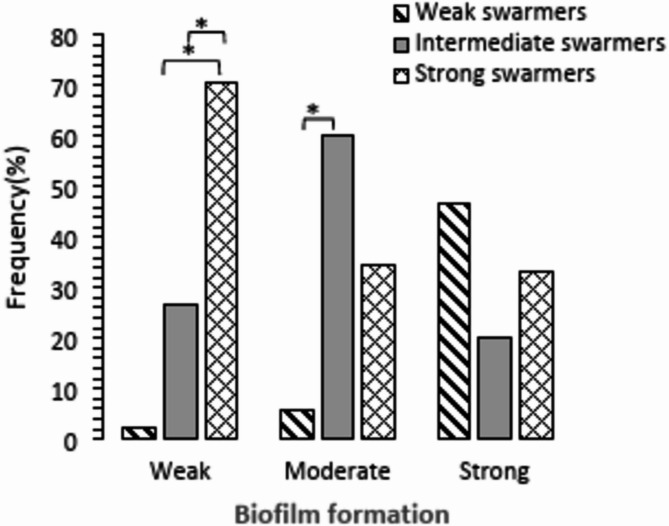
Swarming motility properties of isolates with different biofilm formation ability. *P < 0.001

Chi-square analysis showed that most of the weak biofilm formers (70.7%) had strong swarming motility and they were significantly different from moderate and strong biofilm formers in this regard (Fig. 7).

Optical density values obtained from biofilm formation assessments and swarming values were used for Pearson correlation analysis and findings revealed that biofilm formation was reversely correlated with swarming motility (*p* = 0.01. *r* = − 0.63).

## Discussion

*P. mirabilis* is well known as one of the leading causes of the catheter associated urinary tract infections (CAUTIs). This bacterium could attach urinary catheters and travel along them towards bladder and even kidneys and severely complicate UTI cases [21]. Sabubba et al., reported that swarmer cells of *P. mirabilis* also are able to facilitate moving of non-motile bacteria such as *Klebsiella pneumoniae* on urinary catheters toward infection site [22]. Virulence associated properties of *P. mirabilis* specially biofilm formation ability and swarming motility play critical role in this respect.

The *Proteus mirabilis* equipped with different fimbriae that play role in some virulence associated properties such as biofilm formation at attachment and colonization step of *P. mirabilis* in special cases such as CAUTIs [23]. Data analysis showed *mrpA* gene was significantly more prevalent among moderate and strong biofilm formers in comparison to weak biofilm formers. Other fimbrial genes such as *atfA* and *ucaA* genes were apparently more frequent in strong biofilm formers in comparison to other isolates however, the differences were not statistically significant (Fig. 2). The ATF and UCA fimbriae were known mostly for their association in adhesion to abiotic surface or intestinal epithelium [2]. Majority of studied isolates were obtained for clinical specimens such as urine samples, it may explained different distribution of *mrpA* gene in comparison to *atfA* and *ucaA* genes in isolates with different biofilm formation ability in the present study.

The MR/P fimbriae has recently introduced as most expressed fimbriae of *P. mirabilis* during biofilm formation process in clinical setting [6, 8]. Therefore, superior role of MR/P fimbriae in comparison to other fimbriae in biofilm formation ability of *P. mirabilis* could be hypothesized. It could also be introduced as an attractant target for investigating and designing of novel anti-virulence substances with antibiofilm properties or drug targets. Indeed, some supporting reports about such anti-virulence substances have released before [8, 24].

The *zapA* was most frequent virulence associated gene (100%) among studied virulence associated genes in the present study (Fig. 1). Sun et al., similarly reported *zapA* as the most frequent gene among similar collection of studied virulence genes [25]. The *hlyA* gene had minimum frequency (41.8%) among studied virulence associated genes in *P. mirabilis* isolates (Fig. 1). Similar findings reported recently [4]. Both HlyA and HpmA were known as hemolysins of *P. mirabilis* but higher frequency of *hpmA* gene (97.8%) in comparison to the *hlyA* gene (41.8%) was detected in our findings. Similar finding regarding prevalence of HpmA in comparison to HlyA in *Proteus* isolates was also reported before [26].

Biofilm formation is an important characteristic of many bacteria that support them for surviving in better condition and also protect them from some environmental harsh condition and also immune system activity [27]. In many situations multi-species bacteria could aggregate through mutualistic interactions in a biofilm matrix [28]. Co-aggregation of *P. mirabilis* with other bacteria in the biofilm matrix on urinary-catheters is reported recently [29, 30]. Therefore, production of toxic compounds such as HlyA hemolysin could harm other neighbored bacteria and disturb such aggregations totally. Hence, we thought reverse association of *hlyA* gene with biofilm formation ability that was detected in present study (Fig. 5) is a reasonable finding.

Study findings showed higher frequency of *hlyA* gene among strong swarmers in comparison to the other isolates (Fig. 6). Direct association of hemolysin production with swarming motility that support our findings was reported recently [13].

Scavone et al., have reported that different fimbriae were not associated with swimming and swarming motility in *P. mirabilis* [6]. Similar estimation was found regarding association of four fimbrial genes (*mrpA*, *atfA*, *pmfA* and *ucaA*) with swarming motility in the present study (Fig. 6). In same way, Zonino et al., reported that swimming and swarming motility of *P. mirabilis* isolates was not affected in mutant isolates that don’t have some fimbrial adhesins [31].

Lower frequency of *rsmA* gene among strong swarmer isolates in comparison to the isolates with reduced swarming activity was observed in the present study (Fig. 6). Jen Liaw et al., suggested inhibitory activity of *rsmA* gene on swarming motility and also expression of virulence associated genes in *P. mirabilis* recently [11]. Therefore, it could suggest that *rsmA* gene could suppress swarming motility in *P. mirabilis* isolates.

The present study finding showed most of the strong swarmer isolates (70.7%) had weak biofilm formation capacity and vice-versa condition was detected among weak swarmers (Fig. 7). A reverse correlation was also revealed by Pearson correlation analysis of our data. Therefore, it seems that these two phenomena affect each other antagonistically. Some other studies also support this conclusion, they reported that reversal flagellar rotation, environmental conditions such as surface viscosity and quorum-sensing systems are involved in regulation of surface-associated behaviors such as biofilm formation and swarming motility in a reverse direction [9, 32, 33]. However, in some other studies supporting interaction between swarming and biofilm formation has also hypothesized [34]. Therefore, more complementary studies could help to reach a more trusted conclusion.

## Conclusion

Biofilm formation and swarming motility were investigated in association with diverse virulence associated genes in the present study. The study findings showed that different virulence associated genes play role in different step of *P. mirabilis* infections. Reversal association of biofilm formation with swarming motility that revealed in present study, hypothesized that bacteria may use different fimbrial adhesins for biofilm formation and colonization step, but a bacterial shift from active biofilm producer to a strong swarmer could support bacteria for traveling up to the catheter toward deeper urinary organs. We thought bacteria could overcome bacterial competitors and also immune system cells by production of some toxic substances such as HlyA at this step. However, it has to be investigated with more detailed in future studies. Finally, it seems that *mrpA* and *hlyA* genes could introduced as attractive targets for designing future antibacterial agents or antibiotics.

## Methods

### Bacterial isolates and culture conditions

Ninety-one *P. mirabilis* isolates that were obtained from different clinical samples through a recent study (not published) were used in all experiment. Bacteria were grown in trypticase soy broth with 20% glycerol and kept in −70 °C.

### Biofilm formation assessment

Microtiter plate assay was used for biofilm forming ability assessment. In summary, bacterial suspensions were inoculated into the Mueller-Hinton broth enriched with 1% glucose at final concentration of 5 × 105 CFU/ml in each well of 96-wells flat-bottomed sterile polystyrene microplate. After overnight incubation at 37 °C, microplates were washed with phosphate buffer solution (PBS) and dried. The bacterial biofilms on the microtube walls were stained with 0.1% crystal violet. Subsequently, stained biofilms were washed from microplate walls by 35% acetic acid and were analyzed by microplate reader at 492 nm. Sterile Muller-Hinton broth enriched with 1% glucose was used as control. The ODs higher than OD of control (ODc) were regarded as biofilm producer and they were classified as weak, moderate and heavy biofilm producer if ODc < OD < 2(ODc), 2(ODc) < OD < 4(ODc) and OD > 4(ODc) respectively [35].

### Swarming motility assessment

Bacterial isolates were grown in Moeller-Hinton broth (Conda-Spain) at 37 °C overnight. After duplicate washing with PBS, a 5 µl aliquot of bacterial isolates was inoculated in the center of a dried (at 42 °C for 30 min) Muller-Hinton agar plates and incubate at 37 °C. Swarming motility was measured at 30-minute intervals using a stereo microscope through measuring the distance that bacteria were traveled on the medium from the inoculation point in each time period by a standard caliper. Some other confirmatory properties of swarmer cells such as hyper flagellation and cell elongation also were studied by direct microscopic observation [36]. In brief, we prepared smears from different zone of concentric circles on the agar (swimmer zone and swarmer zone) and they were observed microscopically after Gram staining. Studied isolates were categorized into the three weak, intermediate and strong swarmer groups.

### Bacterial lysate preparation and DNA extraction

Bacterial lysate was prepared through boiling method. In summary, pure colonies of bacterial isolates were harvested by flame sterilized loop and suspended in the 500 µl of distilled water in microtubes. The mixtures were heated at 100 °C for 10 min. In the next step, they were centrifuged (12,000 rpm for 5 min) and then, the supernatant was separated for PCR amplification [37].

### Screening of virulence associated genes

The polymerase chain reaction method was used for detection of virulence associated genes (*rsmA*, *hlyA*, *rsbA*, *mrpA*, *zapA*, *atfA*, *hpmA*, *pmfA*, *ucaA* and *fliL*) using Biometra thermocycler(Germany) and specific primers that were chosen from previous studies (Table 1). The primer sequences were confirmed trough nucleotide BLAST tool (https://blast.ncbi.nlm.nih.gov) and purchased from Bioneer, South Korea. PCR products were separated by electrophoresis on 1% agarose gel (Pharmacia Biotech, Denmark) beside a 100 bp size marker.

**Table 1:** Primer sequences were used for screening of virulence associated genes

VAGs	Primers Sequences (5´→ 3´)	Gene description	Product Size(bp)	Reference
*hlyA*	F: AACAAGGATAAGCACTGTTCTGGCT R: ACCATATAAGCGGTCATTCCCGTCA	HlyA hemolysin subunit	1177	[38]
*ucaA*	F: GTAAAGTTGTTGCGCAAAC R: TTGAGCCACTGTGGATACA	Structural UCA fimbrial protein	560	[25]
*rsmA*	F: TAGCGAGTGTTGACGAGTGG R: AGCGAGGTGAAGAACGAGAA	Repressor of RsmA regulatory system	562	[25]
*rsbA*	F: TTGAAGGACGCGATCAGACC R: ACTCTGCTG TCCTGTGGG TA	Two-component sensor kinase	467	[39]
*hpmA*	F: GTTGAGGGGCGTTATCAAGAGTC R: GATAACTGTTTTGCCCTTTTGTGC	HpmA hemolysin subunit	709	[38]
*zapA*	F: TATCGTCTCCTTCGCCTCCA R: TGGCGCAAATACGACTACCA	IgA-degrading metalloprotease	332	[40]
*mrpA*	F: ACACCTGCCCATATGGAAGATACTGGTACA R: AAGTGATGAAGCTTAGTGATGGTGATGGTGATGAGAGTAAGTCACC	Structural MR/P fimbrial protein	550	[25]
*fliL*	F: CTCTGCTCGTGGTGGTGTCG R: GCGTCGTCACCTGATGTGTC	Regulator of flagellar stator activity	770	[25]
*pmfA*	F: CAAATTAATCTAGAACCACTC R: ATTATAGAGGATCCCTTGAAGGTA	Structural PMF fimbrial protein	810	[25]
*atfA*	F: CATAATTTCTAGACCTGCCCTAGCA R: CTGCTTGGATCCGTAATTTTTAACG	Structural ATF fimbrial protein	537	[25]

### Statistical analysis

The data were introduced into the SPSS software version 26. Chi-square and Fischer’s exact test were used in data analysis. Logistic regression was used for analyzing predictory role of virulence associated genes regarding biofilm formation ability and also swarming motility. The *p* ≤ 0.05 was regarded as significant. Correlation of biofilm formation and swarming motility was analyzed by Pearson correlation analysis.

## Data Availability

The datasets used and/or analyzed during the current study are available from the corresponding author on reasonable request.

## Abbreviations

*Proteus mirabilis*: *P. mirabilis*
MR/P: Mannose Proteus like(MR/P) fimbriae
*PM*F: *Proteus mirabilis *fimbriae
ATF: Ambient temperature fimbriae
UCA: Uroepithelial cell adhesin
VAG: Virulence associated gene
UTI: Urinary tract infection
CAUTI: Catheter associated urinary tract infection
PCR: Polymerase chain reaction
PBS: Phosphate buffer solution
SPSS: Statistical product and service solution software
ANOVA: Analysis of variance
